# Editorial: Community series in immune system disorders: from molecular mechanisms to clinical implications, volume II

**DOI:** 10.3389/fimmu.2026.1807716

**Published:** 2026-02-27

**Authors:** Daniel H. González Maglio, Marisa M. Fernandez, Mauricio C. De Marzi, Ruben D. Motrich

**Affiliations:** 1 Universidad de Buenos Aires, Facultad de Farmacia y Bioquímica, Departamento de Microbiología, Inmunología, Biotecnología y Genética, Instituto de Estudios de Inmunidad Humoral (UBA-CONICET) Consejo Nacional de Investigaciones Científicas y Técnicas (CONICET), Buenos Aires, Argentina; 2 Federation of Clinical Immunology Societies (FOCIS) Center of Excellence Vaccines and Immunotherapies against Infections and Cancers, VITIC, Universidad de Buenos Aires, Buenos Aires, Argentina; 3 Grupo de Investigaciones Básicas yAplicadas en Inmunología y Bioactivos (GIBAIB), Instituto de Ecología y Desarrollo Sustentable (INEDES-CONICET), Luján, Buenos Aires, Argentina; 4 Universidad Nacional de Luján, Departamento de Ciencias Básicas, Luján, Buenos Aires, Argentina; 5 Universidad Nacional de Córdoba, Facultad de Ciencias Químicas, Departamento de Bioquímica Clínica, Centro de Investigaciones en Bioquímica Clínica e Inmunología (CIBICI-CONICET), Córdoba, Argentina; 6 Federation of Clinical Immunology Societies (FOCIS) Center of Excellence Centro de Inmunología Clínica de Córdoba (CICC), Córdoba, Argentina

**Keywords:** immune-endocrine crosstalk, immunomodulation, inflammation, microbiota, mucosal immunity, precision medicine, systemic immunology, translational immunology

**Figure 1:**
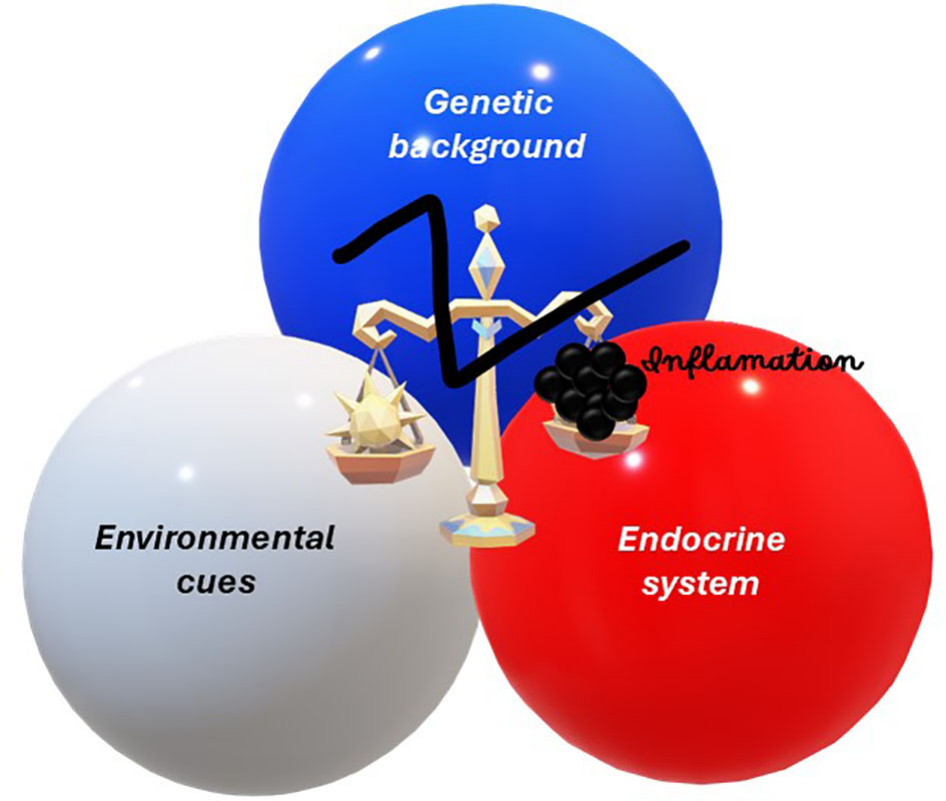
Interplay of genetic, environmental, and endocrine factors in disease etiology.This figure illustrates the balanced interaction among genetic background (blue), environmental cues (white), and endocrine system disruption (red), symbolized by scales tipping toward inflammation (black).

Immunological research has undergone a paradigm shift, moving away from the study of isolated tissues toward a more integrated understanding of systemic networks which are influenced by environmental cues. Modern science now recognizes that the immune system acts as a bridge between genetic predispositions, microbial signals, and endocrine regulation (**Figure 1**). The Community Series in Immune System Disorders: From Molecular Mechanisms to Clinical Implications, volume II, showcases recent breakthroughs in nephrology, rheumatology, oncology, and sexual health. These advances illustrate a complex “multi-organ connectivity”, where systemic inflammation serves as the primary driver of disease onset and/or progression. This editorial explores how these diverse fields converge to redefine precision medicine through the lens of systemic immunology.

In rheumatology, the interplay between the immune system and the endocrine axis is equally profound. In a compelling and novel study, Zhang et al. elucidated key features of the endocrine-immune crosstalk in rheumatoid arthritis (RA), with significant implications for personalized therapeutics. The authors identified global steroid dysregulation in postmenopausal women with RA. Compared to healthy controls, untreated patients exhibited significant suppression of adrenal steroids, including aldosterone, cortisol, and testosterone, alongside an imbalance in estrogen metabolism—specifically a hyperactivated 2-hydroxylation pathway and depleted 16-hydroxylation metabolites. The clinical implications for therapy are striking. Methotrexate treatment unexpectedly exerted hormone-restorative effects, normalizing reduced aldosterone and androgen profiles. In contrast, glucocorticoid therapy paradoxically exacerbated endocrine disruption by further suppressing cortisol and testosterone while pathologically amplifying the 4-hydroxylation pathway, which may contribute to synovial inflammation. These findings show that impaired steroidogenesis and estrogen pathway dysregulation are characteristic features of RA in postmenopausal patients. Moreover, they suggest that long-term glucocorticoid therapy may interfere with their already fragile baseline hormonal homeostasis, highlighting the need of personalized hormonal monitoring.

These *Community Series in Immune System Disorders* also encompasses a pivotal discovery regarding the pathogenesis of cryofibrinogen-associated glomerulonephritis (CF-GN), a nephropathy traditionally considered rare. Through detailed analysis of a clinical case, Tang et al. provide compelling evidence that CF-GN with paraproteinemia should be categorized within the spectrum of monoclonal gammopathy of renal significance (MGRS). This reclassification enables more rational and effective treatment strategies. Their research reveals a fascinating “two-hit” mechanism: the first hit is a genetic predisposition, specifically an intronic mutation in the fibrinogen beta chain gene that increases fibrinogen mRNA expression. However, this mutation often remains asymptomatic until the “second hit” occurs—the accumulation of monoclonal immunoglobulin (MIg). This MIg binds to the variant fibrinogen, forming a mega-complex that precipitates under cold conditions. This complex is not merely a passive byproduct; *in vitro* studies on human mesangial cells show it induces hypertrophy, mitochondrial depletion, and lysosomal degeneration. Furthermore, mice injected with these cryoprecipitates developed not only proliferative glomerulonephritis but also systemic injury in the heart and lungs, including myocardial necrosis and neutrophil infiltration. This underscores the systemic nature of what was once thought to be a localized renal condition.

The series also highlights promising applications of bacterial components as therapeutic adjuvants. Oligopeptidase A (OpdA), a bacterial metalloprotease from *E. coli* and *S. typhimurium*, shows remarkable potential as a tumor vaccine adjuvant. In an elegant study, Silva et al. found that even heat-inactivated OpdA (lacking proteolytic activity) could significantly reduce melanoma metastasis in murine models. Their findings reveal that OpdA acts as a TLR4 agonist, triggering a proinflammatory cascade via the MyD88/TRIF and MAPK signaling pathways. This activation of dendritic cell maturation is characterized by increased expression of co-stimulatory molecules like CD80 and CD86 and the secretion of IL-12 and TNF-α. The resulting immune environment favors a Th1-type adaptive response, increasing tumor-specific CD4+ and CD8+ T lymphocytes that produce IFN-γ. Collectively, these results suggest that bacterial proteases can be repurposed to “flip the switch” in the immunosuppressive tumor microenvironment.

In another study, Shida et al. found that amelogenin (rM180)—a protein traditionally used for periodontal tissue regeneration—has potent local immunosuppressive properties that can be used to improve transplant survival. The authors demonstrated that rM180 can significantly prolong skin allograft survival (by a median of 5.5 days) by modulating the recipient’s immune response. The mechanism involves rM180 being taken up by macrophages, where it inhibits MHC II expression at the transcriptional level by suppressing the transactivator CIITA. This leads to a cascade of local and systemic anti-inflammatory effects: a reduction in Th1 and Th17 cells, and a corresponding increase in regulatory T cells (Treg) and M2-type macrophages. Bioinformatic analysis further identified that rM180 up-regulates genes like *Lcn2* and *Pou2f2*, which negatively regulate the immune response, and *Chil4*, which promotes tissue remodeling. This positioning of amelogenin as a “safe immunosuppressant” without the toxic side effects of conventional drugs offers a new frontier for transplant medicine.

Finally, the series propose the “gut-penis axis” as a definitive model of how distant organ systems are linked by inflammatory signals. Li et al. conducted a comprehensive literature review on mechanistic pathways linking inflammatory bowel disease (IBD) and erectile dysfunction (ED), with a focus on gut-derived inflammation and penile vascular health. The authors synthesized available evidence showing that IBD significantly increases the risk of ED, with prevalence rates as high as 92% in some cohorts. The pathophysiology begins with gut dysbiosis and barrier dysfunction, which allows the translocation of microbial-associated molecular patterns (MAMPs) like LPS into the systemic circulation. This triggers a cytokine storm (TNF-α, IL-1β, IL-6) that impairs penile vascular function by suppressing eNOS expression and inducing reactive oxygen species (ROS) that scavenge nitric oxide (NO). This framework is further enriched by a psychoneuroimmunological perspective. Chronic inflammation can concurrently suppress testosterone synthesis, while psychological stress— common in IBD—activates the HPA axis and sympathetic nervous system, leading to catecholamine surges that promote penile vasoconstriction. Simultaneously, stress reduces vagal tone, impairing the cholinergic anti-inflammatory pathway where acetylcholine normally binds to α7nAChR on macrophages to suppress TNF-α. This holistic view integrates intestinal health, mental health, and vascular homeostasis, suggesting that managing ED in IBD patients requires targeted anti-inflammatory therapies like anti-TNF-α agents or JAK inhibitors alongside traditional PDE5 inhibitors.

The collective insights from these studies reveal that the future of immunology lies in its ability to bridge gaps between seemingly unrelated systems. Whether it is a genetic “hit” in a kidney gene activated by a monoclonal protein, a bacterial protease modulating melanoma, or an intestinal leak causing vascular damage in the penis, the common denominator is a systemic immune-inflammatory response. By identifying these shared pathways and mechanisms, medicine can move toward more integrated, multidisciplinary management strategies that treat the patient as a cohesive biological network.

